# Glycoconjugate Vaccine Containing *Escherichia coli* O157:H7 O-Antigen Linked with Maltose-Binding Protein Elicits Humoral and Cellular Responses

**DOI:** 10.1371/journal.pone.0105215

**Published:** 2014-08-19

**Authors:** Zhongrui Ma, Huajie Zhang, Wenjing Shang, Faliang Zhu, Weiqing Han, Xueer Zhao, Donglei Han, Peng George Wang, Min Chen

**Affiliations:** 1 School of Life Sciences and The State Key Laboratory of Microbial Technology, National Glycoengineering Research Center, Shandong University, Jinan, Shandong, China; 2 Department of Biochemistry and Molecular Biology, Binzhou Medical University, YanTai, Shandong, China; 3 Department of Immunology, Shandong University School of Medicine, Jinan, Shandong, China; 4 College of Pharmacy and the State Key Laboratory of Medicinal Chemical Biology, Nankai University, Tianjin, China; Imperial College London, United Kingdom

## Abstract

Glycoconjugate is one of the most efficacious and safest vaccines against bacterial pathogens. Previous studies of glycoconjugates against pathogen *E. coli* O157:H7 focused more on the humoral responses they elicited. However, little was known about their cellular responses. In this study, we exploited a novel approach based on bacterial protein N-linked glycosylation system to produce glycoconjugate containing *Escherichia coli* O157:H7 O-antigen linked with maltose-binding protein and examined its humoral and cellular responses in BALB/c mice. The transfer of *E. coli* O157:H7 O-antigen to MBP was confirmed by western blot and MALDI-TOF MS. Mice injected with glycoconjugate O-Ag-MBP elicited serum bactericidal antibodies including anti-*E. coli* O157:H7 O-antigen IgG and IgM. Interestingly, O-Ag-MBP also stimulated the secretion of anti-*E. coli* O157:H7 O-antigen IgA in intestine. In addition, O-Ag-MBP stimulated cellular responses by recruiting Th1-biased CD4^+^ T cells, CD8^+^ T cells. Meanwhile, O-Ag-MBP induced the upregulation of Th1-related IFN-γ and downregulation of Th2-related IL-4, and the upregulation of IFN-γ was stimulated by MBP in a dose-dependent manner. MBP showed TLR4 agonist-like properties to activate Th1 cells as carrier protein of O-Ag-MBP. Thus, glycoconjugate vaccine *E. coli* O157:H7-specific O-Ag-MBP produced by bacterial protein N-linked glycosylation system was able to elicit both humoral and Th1-biased cellular responses.

## Introduction

Enterhemorrhagic *Escherichia coli* (EHEC) O157:H7, as a severe enteric pathogen in human, generally causes bloody diarrhea, hemorrhagic colitis and hemolytic uremic syndrome (HUS) [1], [2]. Conventional antibiotic therapy increases the incidence of HUS due to the release of Shiga toxin into intestinal mucosa by antibiotic-mediated bacteriolysis [3], [4]. There is an urgent need for vaccines to prevent *Escherichia coli* (*E. coli*) O157:H7 infection. Several potential vaccines have been tested in a number of preclinical or clinical studies [5].

Glycoconjugate (O-antigen covalently linked to carrier protein) is considered one of the most efficacious and safest vaccines against bacterial pathogens. O-antigen (O-Ag) plus core oligosaccharides and lipid A form an intact lipopolysaccharide (LPS) of gram-negative bacteria. O-Ag is exposed on the very outer surface of the bacterial cell, and, as a consequence, is a target for recognition by host antibodies. Even though, O-Ag is T-lymphocyte independent antigen like other bacterial polysaccharides. O-Ag alone cannot induce a high serum IgG and sustained T cell memory [6].

Carrier protein contributes a lot to glycoconjugate's immunological efficacy. When O-Ag is conjugated to carrier protein, this glycoconjugate could elicit high serum IgG antibodies to the O-Ag component and induce memory B cells and long-lived T cell memory [7]. The common used carrier proteins in glycoconjugate vaccines include tetanus toxoid, diphtheria toxoid, CRM197, rEPA (*Pseudomonas aeruginosa* exotoxin A), KLH (keyhole limpet hemocyanin) and flagellin [8]. Currently, exploration of new carrier proteins focuses on T cell epitopes, Toll-like receptors (TLRs), etc. [9]. Recent studies showed that *E. coli* maltose-binding protein (MBP) had TLR4 agonist-like properties to induce the activation of NF-κB signaling pathway and secretion of proinflammatory cytokines like IL-1β, IL-6, IL-8, TNF-α and IL-12p70 [10]. Some bioactive proteins such as MUC1 and BCG, after fused to MBP, showed better immunity against tumors [11], [12], indicating that MBP could be ideal carrier protein in anti-tumor vaccines. However, little was known about the potential application of MBP against pathogenic bacteria and its immunological enhancement as carrier protein towards glycoconjugates.

Previous studies showed that glycoconjugates including O-Ag-rEPA [13] and O-Ag-Stx [14] against *E. coli* O157:H7 were able to induce IgG and IgM with serum bactericidal activity. Even though, little was known about the expressions of T-cell differentiation and corresponding secretion of cytokines in response to these glycoconjugates. There is increasing evidence for the role of cellular responses in protection, cellular responses are recognized as more persistent than antibodies [15].

Traditional chemical technologies to produce glycoconjugates are complex and go through multi-step processes. In recent years, via bacterial protein N-linked glycosylation system from *Campylobacter jejuni* (*C. jejuni*), next generation of glycoconjugate vaccines is booming rapidly. Oligosaccharyltransferase (OTase) PglB, the key enzyme of *C. jejuni*, is able to transfer glycan with N-acetyl at the reducing end sugar to asparagine (N) residue of consensus sequence D/E-X-N-Y-S/T (X, Y≠proline) [16], [17]. Currently, using this convenient in vivo method, O-Ag from a variety of strains including *E. coli* O121 [18], *Yersinia enterocolitica* O9 [19] and *Francisella tularensis*, [20] were coupled to diverse carrier proteins. In this study, we evaluated whether O-Ag from *E. coli* O157:H7 was a suitable substrate for PglB and explored to use this in vivo protein glycosylation platform to generate a novel glycoconjugate O-Ag-MBP against *E. coli* O157:H7.

In this study, we provided a novel way to produce anti-*E. coli* O157:H7 glycoconjugate O-Ag-MBP and showed a comprehensive profile of immune system induced by O-Ag-MBP. Meanwhile, we explored the potential role of MBP as a novel carrier protein of glycoconjugate vaccines against pathogenic bacteria to enhance the immunological efficacy of O-Ag-MBP.

## Materials and Methods

### Ethics statement

All animal studies were approved by the Ethics Committee of Shandong University School of Medicine (No. 001 in 2011 for Animal Ethics Approval) and all efforts were made to minimize sufferings.

### Bacterial strains and growth conditions

*E. coli* DH5α was used for cloning of plasmids. *E. coli* CLM24 was used for glycoconjugate production experiments. CLM24 derived from *E. coli* W3110 by knocking out of *waal* gene (performed in this study). *E. coli* strains were grown in Luria-Bertani (LB) broth unless otherwise noted. For glycoconjugate production experiments, *E. coli* strains were grown in Terrific Broth (TB) broth. Ampicillin at 100 µg/ml, chloramphenicol at 25 µg/ml and spectinomycin at 50 µg/ml were used for plasmids selection as needed.

### Plasmids construction

The plasmid pBAD24-MBP-GT-6×-His was constructed by inserting *malE* gene from plasmid pMAL-p5x (New England Biolabs), DNA encoding the O-Ag acceptor peptide tag GT (i.e., N-DQNATGGDQNATGGDQNATGGDQNAT-C) and a tag 6×-His (i.e., N-HHHHHH-C) between SmaI and SalI of pBAD24 (Induced by L-arabinose). The plasmid pACT3-PglB was constructed by inserting *pglb* gene from *C. jejuni* NCTC 11168 between SmaI and SalI of pACT3 (Induced by IPTG). The plasmid pYES1L-O-Ag was constructed by inserting *rfb* gene cluster and genes located upstream (including *wcaM*, *z3206*, *galF*) and downstream (including *gnd*, *ugd*, *wzz*, *hisl*) of *rfb* cluster (Figure S1) from *E. coli* O157:H7 into plasmid pYES1L (Self-expression without inducing) using GENEART High-Order Genetic Assembly System (Invitrogen).

### LPS sliver-stained SDS-PAGE

The LPS profile of the proteinase K-digested whole cells was examined by sliver-stained SDS-PAGE. Briefly, pellets from 1 ml *E. coli* broth with OD_600_ of 1.0 was resuspended in 50 µl 1×loading buffer and boiled for 6 min. After cooling to room temperature, 80–100 µg Proteinase K (Thermo Scientific) was added and then incubated at 60°C for 2 hrs. 10 µl sample was load onto 12% SDS-PAGE, and then sliver stained as previously described [21].

### Expression and purification of O-Ag-MBP

CLM24 transferred with pBAD24-MBP-GT-6×-His, pACT3-PglB and pYES1L-O-Ag was grown in 50 ml LB broth at 37°C for 16 hrs, with shaking. Cultures were then inoculated 1/100 into 1 L TB broth and further grown at 37°C with shaking until OD_600_ reached 0.6. Subsequently, 0.1% (w/v) L-arabinose and 50 µM IPTG were added to induce the expression of MBP and PglB, respectively. After another incubation at 28°C for 6 hrs, 0.1% (w/v) L-arabinose was added again for further induction of MBP.

After a total 20-hr induction at 28°C, cells were harvested by centrifugation at 10,000 g for 10 min and the periplasmic component of the cells were extracted by lysozyme treatment (20 mM Tris-HCl (pH 7.5), 20% (w/v) sucrose, 1 mM EDTA, 1 mg/ml lysozyme, at 4°C for 1 hr). Then, after centrifugation at 10,000 g for 30 min, cell debris was removed and the supernatant was loaded to a Ni-NTA column filled with 3 ml Ni-NTA agarose (GE Healthcare), which was pre-equilibrated with wash buffer (10 mM imidazole, 0.5 M NaCl, 20 mM Na_2_HPO_4_/NaH_2_PO_4_ buffer, pH 7.4). Subsequently, the column was washed with wash buffer again and then eluted with elution buffer (250 mM imidazole, 0.5 M NaCl, 20 mM Na_2_HPO_4_/NaH_2_PO_4_ buffer, pH 7.4). Fraction containing the purified glycoconjugate was collected and then desalted using centrifugal filter (Amicon Ultra-15, Milipore) against PBS (PH 7.4).

### Protein and carbohydrate quantification of O-Ag-MBP

The protein quantity of O-Ag-MBP was measured using Bradford method. The carbohydrate quantity of O-Ag-MBP was measured using phenol-sulfate method. Briefly, 80 µl sample diluent, 40 µl 5% (v/v) aqueous phenol and 200 µl concentrated H_2_SO_4_ were consecutively mixed in an Eppendorf tube. After reaction at 30°C for 30 min, read against glucose standards at OD_490_.

### Western blot analysis

Samples were separated by 12% SDS-PAGE gels and transferred onto polyvinylidene fluoride (PVDF) membranes. The PVDF membranes were probed with one of the following: anti-MBP antibody (TransGen Biotech, China), anti-6×-His antibody (Beyotime, China) and O157:H7 antiserum. In case of anti-MBP antibodies and anti-6×-His antibodies, HRP-rabbit anti-mouse IgG (Invitrogen) was used as the secondary antibody. In case of O157:H7 antiserum, HRP-goat anti-rabbit IgG (Invitrogen) was used as the secondary antibody.

### MALDI-TOF MS analysis

1 µl sample was pipetted onto a spot on the MALDI-TOF steel target plate. After the sample had dried, 2 µl of the SA matrix (50% ACN, 50% H_2_O, 0.1% TFA) was pipetted onto the samples. After the matrix had dried, spectra were acquired over a mass/charge (m/z) ratio of 3,500 to 80,000 using a MALDI-TOF mass spectrometer (AXIMA Confidence, SHIMAZU) in linear mode and power 130.

### LPS extraction from *E. coli* O157:H7

LPS was extracted from *E. coli* O157:H7 using the hot phenol-water method as previously described [22]. Briefly, 10 g dried *E. coli* O157:H7 cells were extracted with 100 ml 50% (v/v) aqueous phenol at 65°C for 20 min. After centrifugation at 10,000 g for 30 min, the cell debris was discarded and the top aqueous solution was dialyzed against ddH_2_O to remove phenol. Then the solution was lyophilized and then dissolved in 10 ml ddH_2_O. Subsequently, the solution was consecutively treated with DNase I, RNase A and Proteinase K (Thermo Scientific) according to the manufacturer's instructions. After ultracentrifugation at 110,000 g for 4 hrs, the precipitated gels were dissolved in ddH_2_O and lyophilized to obtain pure LPS.

### BALB/c mice immunization

6-week-old female BALB/c mice were divided into three groups, each group contained 8 mice. Mice of each group were injected subcutaneously for once immunization with one of the following: 750 µl of 30 µg (protein quantity) O-Ag-MBP in PBS (pH 7.4) +750 µl adjuvant, 750 µl of 30 µg (protein quantity) MBP in PBS+750 µl adjuvant and 750 µl PBS+750 µl adjuvant, respectively. Mice were injected three times of a two-week-interval. The first immunization used Freund's Complete Adjuvant (FCA) (Sigma) and the second and third immunizations used Freund's Incomplete Adjuvant (FIA) (Sigma). Seven days after the third immunization, the blood were taken from the tail vein of mice and centrifuged at 3,000 g for 30 min to obtain sera. 100 mg feces were taken from mice and immediately mixed with 1 ml PBS (pH 7.4), the supernatants were collected after centrifugation at 10,000 g for 30 min. The mice were then killed and the splenic mononuclear cells were isolated using mice lymphocyte separation liquid (Dakewe Biotech Co., China) and counting.

### Antibody titer assay

Immunoglobulin subclass titer assay of sera and feces were measured by ELISA as described previously [19]. Briefly, the 96-well plate (Costar Polystyrene High Binding Plate 3590) was coated with 100 µl of 1 µg/ml extracted *E. coli* O157:H7 LPS in 0.05 M Na_2_CO_3_ (pH 9.8) at 4°C overnight. The coated plate was then washed with 250 µl PBS+ Tween (PBST) (pH 7.4) for three times and then blocked with 2% (w/v) BSA in PBST for 2 hrs at room temperature. After washed with another 250 µl PBST for 3 times, the plate was incubated with 100 µl of sera or feces diluted with PBS at room temperature for 2 hrs. After washed with 250 µl PBST for 5 times, HRP-goat anti-mouse IgG, IgM or IgA (Abcam) was added to the plate at a dilution of 1∶20,000 in PBS for 1 hr at room temperature. After washed with another 250 µl PBST for 5 times, 100 µl TMB solution was added to the plate and incubated for 15 min before 100 µl 1 M HCl was added. The plate was then read at OD_450_.

MBP-specific IgG titer assay was measured by ELISA according to immunoglobulin subclass titer assay. The difference is, 5 µg/ml MBP in 0.05 M Na_2_CO_3_ (pH 9.8) was used as the coating antigen.

*E. coli* O157:H7-specific IgG subtypes titer assay was measured by ELISA according to immunoglobulin subclass titer assay. The difference is, HRP-goat anti-mouse IgG1, IgG2a or IgG2b (Abcam) were used as the second antibodies.

### Cytokine quantification assay

Sera of mice were analyzed for IFN-γ and IL-4 by cytokine ELISA kit (Dakewe Biotech Co., China). ELISA was performed according to the manufacturer's instructions.

### ELISPOT assay

IFN-γ and IL-4 secretions by the splenic mononuclear cells were analyzed using precoated Enzyme-Linked Immunospot (ELISPOT) kit (Dakewe Biotech Co., China). ELISA was performed according to the manufacturer's instructions. Spots were analyzed with a Biosys Bioreader 4000 (Bio-Sys GmbH, Germany).

### Flow cytometry assay

The splenic mononuclear cells (1×10^6^ cells) were fixed with paraformaldehyde for 1 hr and washed twice with FACS solution (PBS containing 2% FCS and 0.1% NaN_3_). The cells were then incubated with cell surface antibodies (FITC-CD3, PE-CD4, PE-CD8) at 4°C for 30 min in the dark, and then washed twice with FACS solution. Subsequently, the stained cells were analyzed with a FACS flow cytometer (Beckman Coulter FC500, USA).

### Bactericidal activity assay

Briefly, 50 µl serum dilutions with PBS (pH 7.4) from 1∶20 to 1∶1,280 were added to 96-well plate (Costar Polystyrene High Binding plate 3590), then mixed with 50 µl of ∼10^4^/ml *E. coli* O157:H7 bacteria in 1% peptone supplemented with 5% rabbit sera. The plate was incubated at 37°C for 1 hr. After that, 10 µl CCK8 was added and plate was further incubated at 37°C for 4 hrs. The plate was then read at OD_450_.

### Statistical analysis

All figures and statistical analyses were generated using the program GraphPad Prism version 5.0. Data were shown as means ± standard deviation (SD). *t* test was used for analyses when two groups were compared. One-way ANOVA was used to test for statistical significance of differences between three experimental groups.

## Results

### Expression of *E. coli* O157:H7 O-Ag in *E. coli* W3110

The O-Ag biosynthesis pathway of *E. coli* O157:H7 is a process involving multiple enzymes. The assembly, transport and polymerization of O-Ag is encoded by *rfb* gene cluster and the chain length is determined by *wzz* gene (Figure S1). A recombinant plasmid pYES1L-O-Ag responding for *E. coli* O157:H7 O-Ag biosynthesis with its native promoter was constructed in this study.

**Figure 1:**
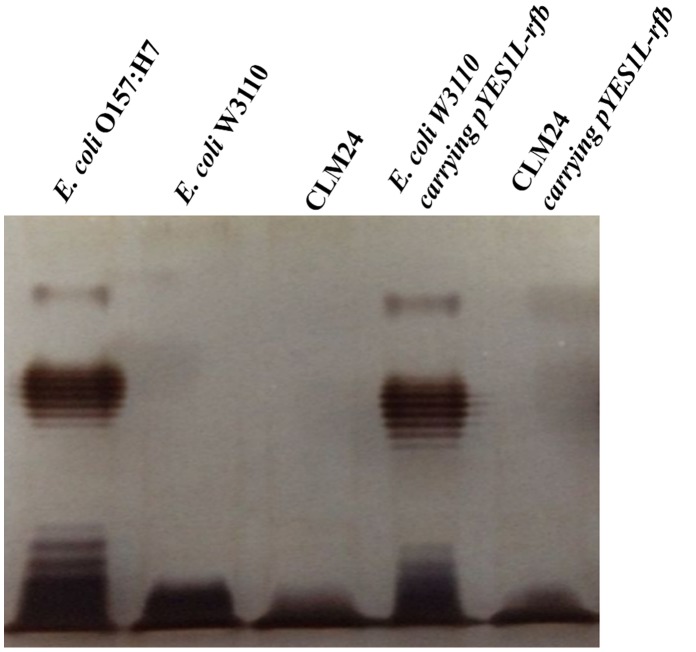
Sliver-stained SDS-PAGE of proteinase K-digested cell lysates.

To confirm the expression of *E. coli* O157:H7 O-Ag in host strain *E. coli* W3110, the LPS profiles of the strains were compared. The silver-stained LPS profile of proteinase K-digested cell lysates of *E. coli* W3110 containing pYES1L-O-Ag showed similarity with that of *E. coli* O157:H7 (Figure 1). Corresponding bands were absent in vector-deficient *E. coli* W3110 cells (Figure 1). In consideration that O-Ag is dominant in forming heterogeneous LPS profiles, and *E. coli* W3110 cannot synthesize its own O-Ag due to the inactivation of *wbbl* gene [23], the similar LPS profiles between *E. coli* W3110 containing pYES1L-O-Ag and that of *E. coli* O157:H7 verified the expression of *E. coli* O157:H7 O-Ag in *E. coli* W3110 and the chain length was controlled mainly by *wzz*_O157_ gene instead of *wzz*_W3110_ gene.

### OTase PglB transfers *E. coli* O157:H7 O-Ag to acceptor protein MBP

To block the formation of LPS and redirect O-Ag covalently linking to acceptor protein under the catalysis of OTase PglB, *waal* gene encoding for the ligase WaaL (in charge of linking O-Ag to core oligosaccharides-lipid A) was knocked out from *E. coli* W3110 chromosome to generate CLM24. The LPS profile of CLM24 containing pYES1L-O-Ag showed no bands responsible for *E. coli* O157:H7 LPS, with *E. coli* W3110 containing pYES1L-O-Ag as control (Figure 1).

**Figure 2:**
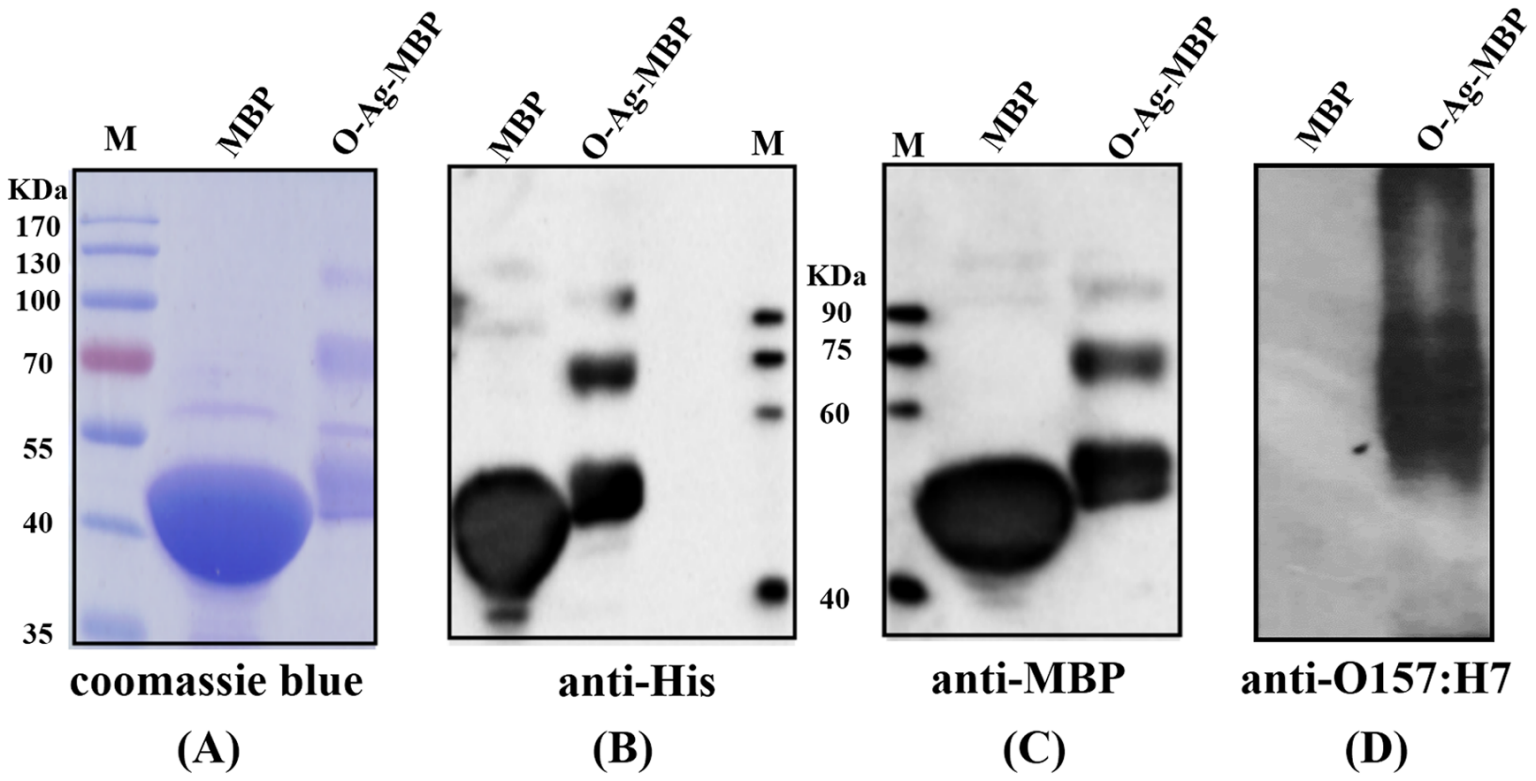
Glycosylation of MBP with *E. coli* O157:H7 O-Ag and analyzed by (A), coomassie blue staining and western blot with (B), anti-6×-His antibody; (C), anti-MBP antibody; (D), O157:H7 antiserum.

**Figure 3:**
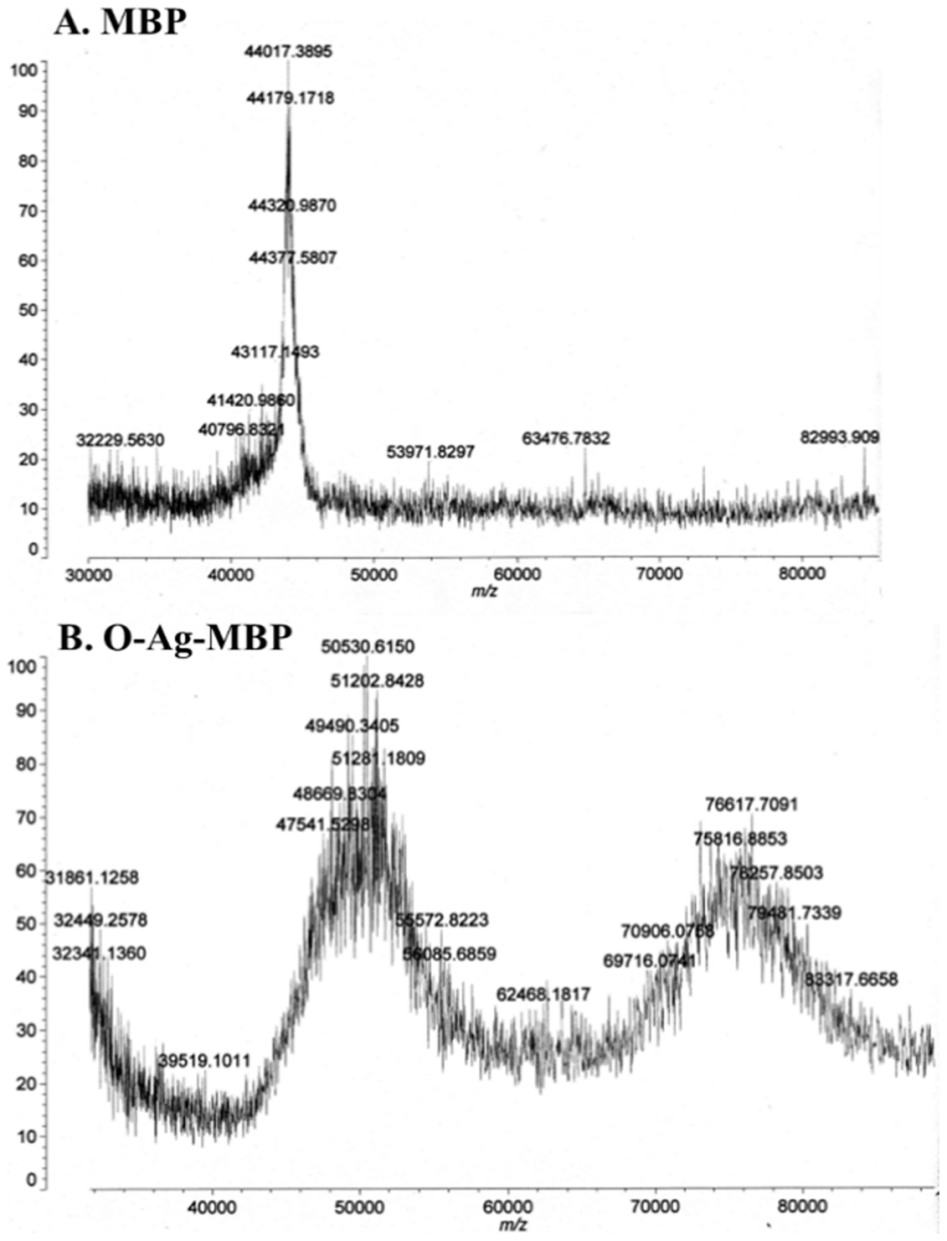
MALDI-TOF MS analysis of (A) MBP and (B) O-Ag-MBP. (**A**), The peak with m/z 44017.39 corresponds to un-glycosylated MBP; (**B**), The peak with m/z 50530.62 and m/z 76617.71 correspond to glycosylated MBP (O-Ag-MBP).

Next, in order to transfer *E. coli* O157:H7 O-Ag to MBP, three recombinant plasmids pACT3-PglB, pBAD24-MBP-GT-6×-His and pYES1L-O-Ag were co-transformed into host strain CLM24. The recombinant cells were grown and induced as described previously. The purified fractions were separated by SDS-PAGE and then visualized by coomassie blue and western blot. By coomassie blue staining, the purified fraction from CLM24 cells carrying pBAD24-MBP-GT-6×-His and pYES1L-*rfb* (strain 1) showed a major band with a mass of ∼45 KDa (Figure 2). By further MALDI-TOF MS analysis, molecular weight of the band of strain 1 was 44017 Da (Figure 3A). By contrast, the purified fraction from CLM24 cells carrying pBAD24-MBP-GT-6×-His, pYES1L-O-Ag and pACT3-PglB (strain 2) showed two main ladders of bands with mass range near ∼50 KDa and ∼70 KDa (Figure 2). By further MALDI-TOF analysis, these two ladders of bands of strain 2 peaked at 50530 Da and 76617 Da, respectively (Figure 3B). Thus, we hypothesized that the band in strain 1 with a mass of ∼45 KDa was MBP and these higher-molecular-mass bands in strain 2 were glycosylated forms of MBP. To confirm this, we tested the interactions of the purified fractions with anti-MBP antibody, anti-6×-His antibody and O157:H7 antiserum, respectively. The band with a mass of ∼45 KDa in strain 1 reacted with anti-MBP antibody and anti-6×-His antibody, but not O157:H7 antiserum (Figure 2), suggesting that the band of strain 1 was un-glycosylated form of MBP. These higher-molecular-mass bands of strain 2 reacted with O157:H7 antiserum, together with anti-MBP antibody and anti-6×-His antibody (Figure 2), suggesting that they were glycosylated forms of MBP, i.e., O-Ag-MBP.

In shake-flask fermentation of 1 L TB broth, MBP purified from strain 1 was counted for 0.53 mg (protein quantity) and 0.02 mg (carbohydrate quantity). O-Ag-MBP purified from strain 2 was counted for 0.85 mg (protein quantity) and 0.36 mg (carbohydrate quantity). So in O-Ag-MBP, the weight of O-Ag component was calculated to 0.38 mg per mg MBP. Consideration that the molecule weight of one repeat unit of *E. coli* O157:H7 O-Ag (-D-PerNAc-α-1,3-L-Fuc-α-1,4-Glc-β-1,3-GalNAc-α- [24]) was 769 Da and MBP was 44017 Da, we calculated the average number of O-Ag repeat units linked to one MBP in purified O-Ag-MBP was about 22.

### Humoral responses in BALB/c mice immunized with O-Ag-MBP

To evaluate the potential use of O-Ag-MBP as glycoconjugate vaccine, O-Ag-MBP was injected subcutaneously into BALB/c mice to measure their immune responses, with MBP and PBS as controls.

**Figure 4:**
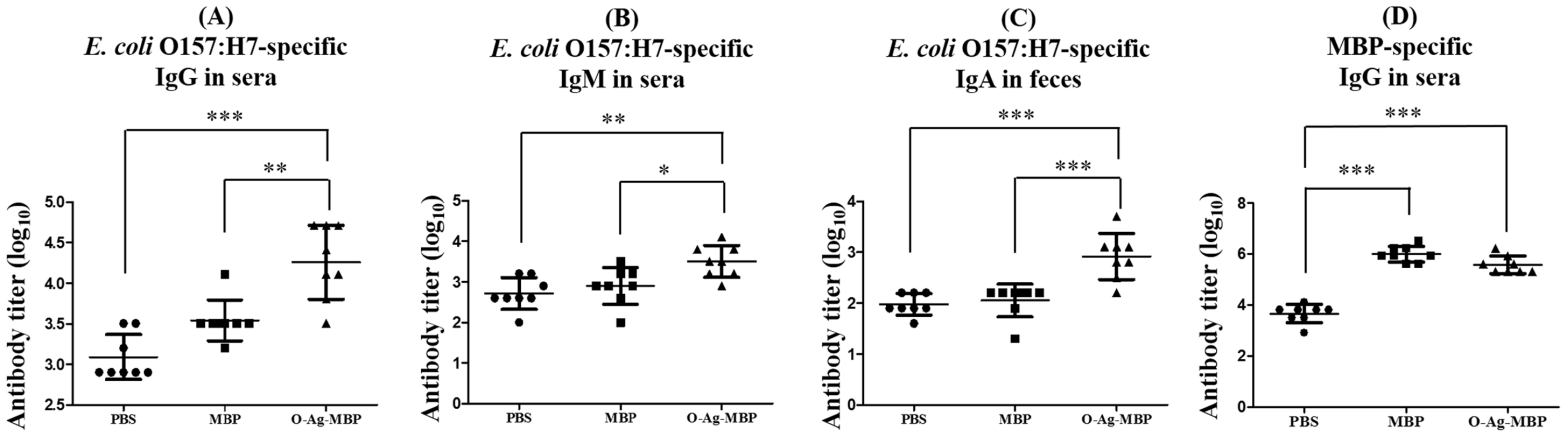
Antibody responses after third immunization. (**A**), *E. coli* O157:H7-specific IgG antibody titers of serum samples; (**B**), *E. coli* O157:H7-specific IgM antibody titers of serum samples; (**C**), *E. coli* O157:H7-specific IgA antibody titers of feces samples; (**D**), MBP-specific IgG antibody titers of serum samples. The cutoff value was OD_negative control_×2.1. Results were expressed as the arithmetic mean ±SD indicated by error bars. Differences of two groups were indicated with symbols (*: P<0.05; **: P<0.01; ***: P<0.001).

Seven days after the third immunization, sera and feces obtained from mice were analyzed for *E. coli* O157:H7-specific immunoglobulin subclass titers by ELISA, using extracted *E. coli* O157:H7 LPS as a coating antigen. As expected, mice immunized with O-Ag-MBP elicited higher serum total IgG compared with mice immunized with MBP (P<0.01) and PBS (P<0.001), respectively (Figure 4A). Similarly, only O-Ag-MBP group elicited a rise in serum IgM compared with mice immunized with MBP (P<0.05) and PBS (P<0.01), respectively (Figure 4B). Interestingly, IgA secretions in feces were detected higher in O-Ag-MBP group compared with MBP group (P<0.001) and PBS group (P<0.001) (Figure 4C), reflecting that O-Ag-MBP stimulated the secretion of IgA in intestine. O-Ag-MBP group had significant differences compared with PBS and MBP group, both of which also had a detectable background responses towards *E. coli* O157:H7. One reason might be that the mice accessed to *E. coli* O157:H7 or *E. coli* O157:H7-like bacteria before or in the experiments.

We also measured MBP-specific IgG titers using purified MBP from strain 1 as the coating antigen. Approximately the same level of MBP-specific IgG was measured in serum of MBP group and O-Ag-MBP group (Figure 4D), suggesting that MBP-specific IgG was produced after immunizing with either MBP or O-Ag-MBP.

### O-Ag-MBP induces killing of *E. coli* O157:H7

**Figure 5:**
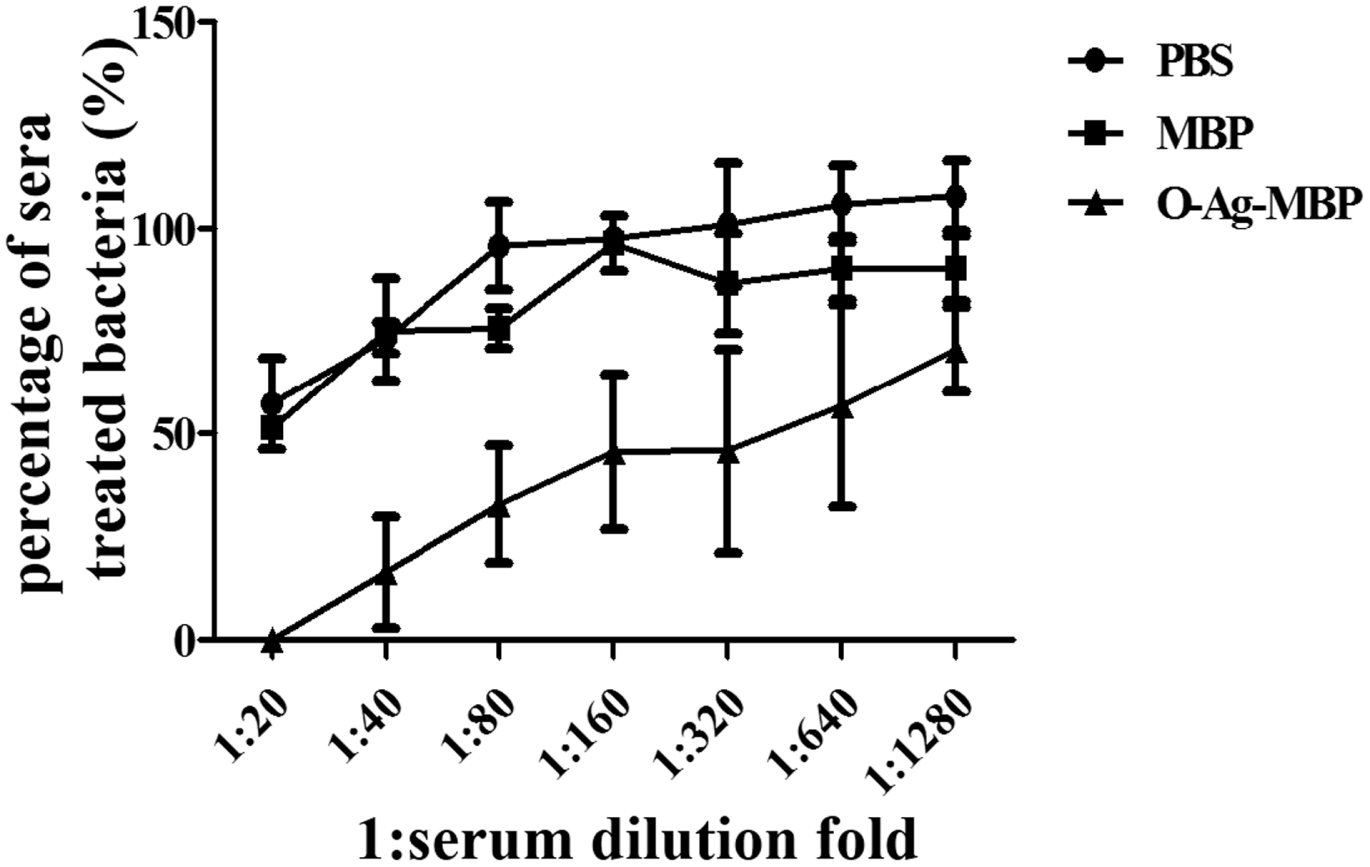
Serum bactericidal activity against *E. coli* O157:H7. The % killing of *E. coli* O157:H7 are expressed as the arithmetic mean ±SD indicated by error bars.

To evaluate serum bactericidal activity of O-Ag-MBP against *E. coli* O157:H7, diluted serum samples were incubated with *E. coli* O157:H7 in rabbit sera and then developed by CCK8, which allows sensitive colorimetric assay for the determination of cell viability. The highest serum dilution fold yielding 50% killing of *E. coli* O157:H7 in O-Ag-MBP group was almost 320, while in MBP group and in PBS group were almost 20 (Figure 5). When the serum dilution fold of O-Ag-MBP was 20, almost all of bacteria were killed (Figure 5). These results suggested that the sera in O-Ag-MBP group showed obvious bactericidal activity against *E. coli* O157:H7.

### Cellular responses in BALB/c mice immunized with O-Ag-MBP

**Figure 6:**
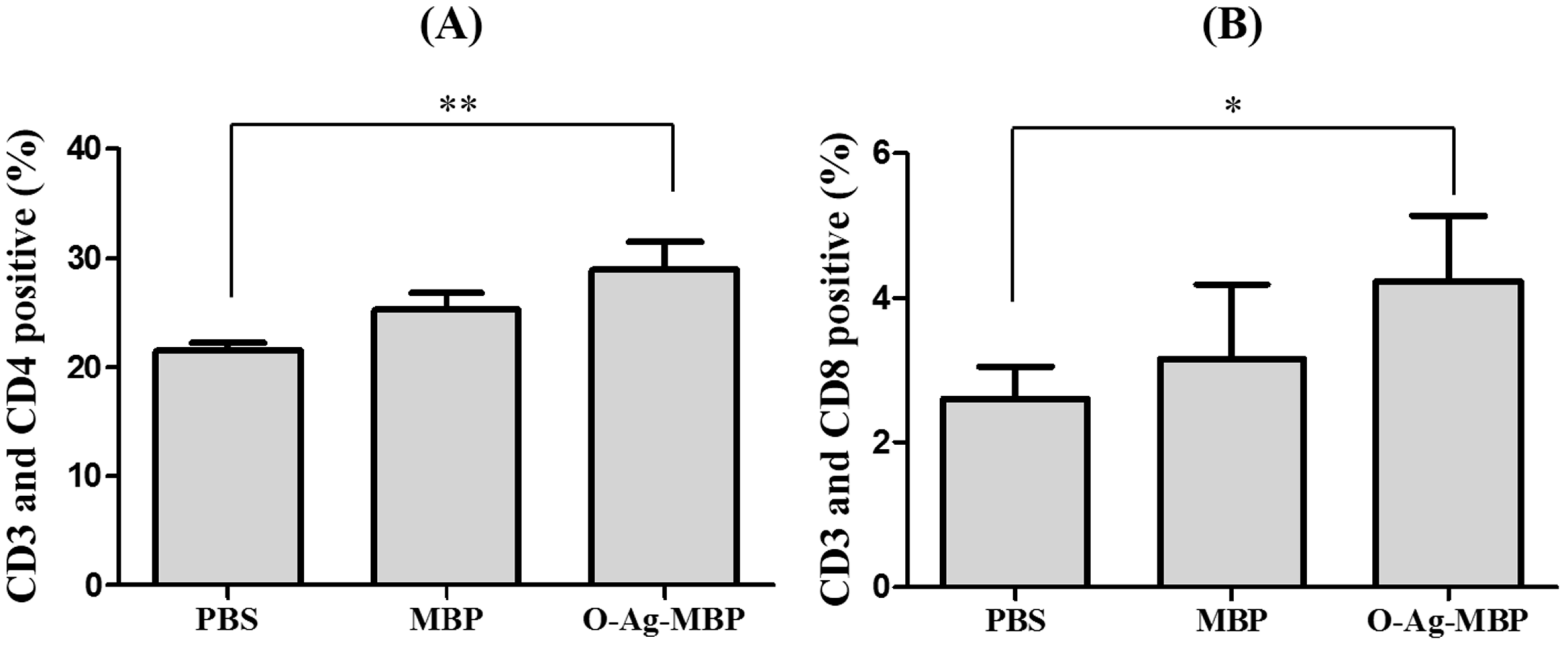
Spleen lymphocyte subsets assay. Spleen lymphocytes were isolated and stained for FITC-CD3, PE-CD4 or PE-CD8 antibodies and then analyzed by flow cytometry. The percentage of (**A**), CD3^+^ CD4^+^ T cells; (**B**), CD3^+^ CD8^+^ were expressed as the arithmetic mean ±SD indicated by error bars. Exprements were repeated three times. Differences of two groups are indicated with symbols (*: P<0.05; **: P<0.01).

To reveal immunization-associated changes in T lymphocyte subsets, Seven days after the third immunization, splenic mononuclear cells were collected and then stained with cell marker antibodies specific for CD3, CD4 or CD8 T cells, and then measured by flow cytometry. The upregulation in T-cell expression of CD3^+^CD4^+^ surface antigens associated with active immunization were more significant with O-Ag-MBP than with PBS (P<0.01) (Figure 6A). Meanwhile, the upregulation of CD3^+^CD8^+^ antigens were significant in O-Ag-MBP group than in PBS group (P<0.05) (Figure 6B). These results indicated that O-Ag-MBP stimulated the proliferation of CD4^+^ and CD8^+^ T cells.

**Figure 7:**
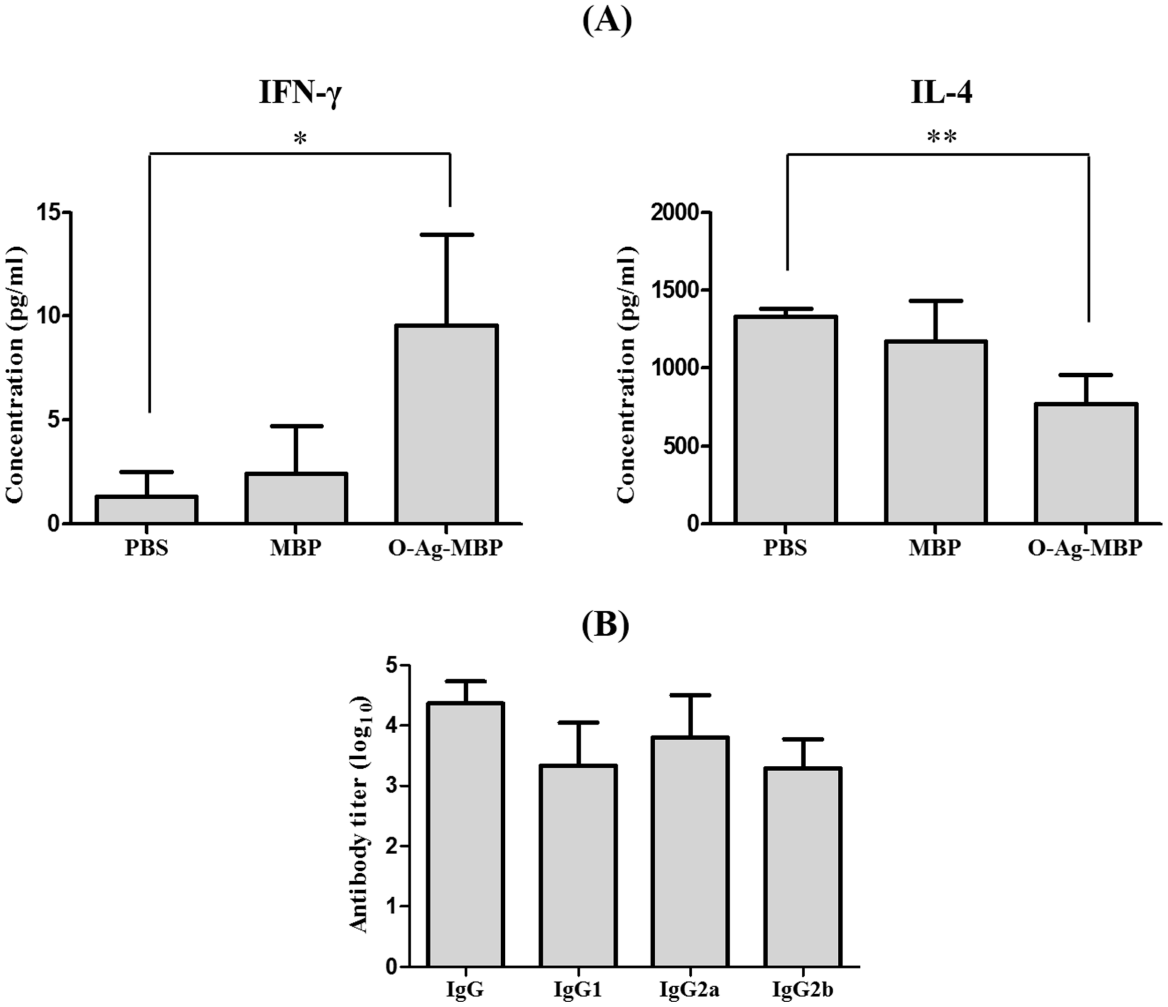
Induction of Th1-biased responses by O-Ag-MBP. (**A**), Secretion of IFN-γ and IL-4 in serum samples of immunized mice. Differences of two groups are indicated with symbols (*: P<0.05; **: P<0.01). (**B**), *E. coli* O157:H7-specific IgG subtypes induced by O-Ag-MBP. BALB/c mice were immunized with O-Ag-MBP and serum samples were collected and *E. coli* O157:H7-specific IgG, IgG1, IgG2a and IgG2b titers were determined by ELISA. The cutoff value was OD_negative control_×2.1. Results were expressed as the arithmetic mean ±SD indicated by error bars.

T cell responses were further assessed by measurement of cytokines in sera. Immunization of mice with O-Ag-MBP induced T cells that secreted higher concentrations of IFN-γ than with PBS (P<0.05) (Figure 7A) and low concentrations of IL-4 than with PBS (P<0.01) (Figure 7A), indicating that O-Ag-MBP induced Th1-biased response. By IgG subtypes titer assay (Figure 7B), we found that IgG2a accounts for the main type of IgG, which also indicating that O-Ag-MBP induced mainly Th1-biased response.

### Immunological enhancement of MBP as carrier protein towards O-Ag-MBP

**Figure 8:**
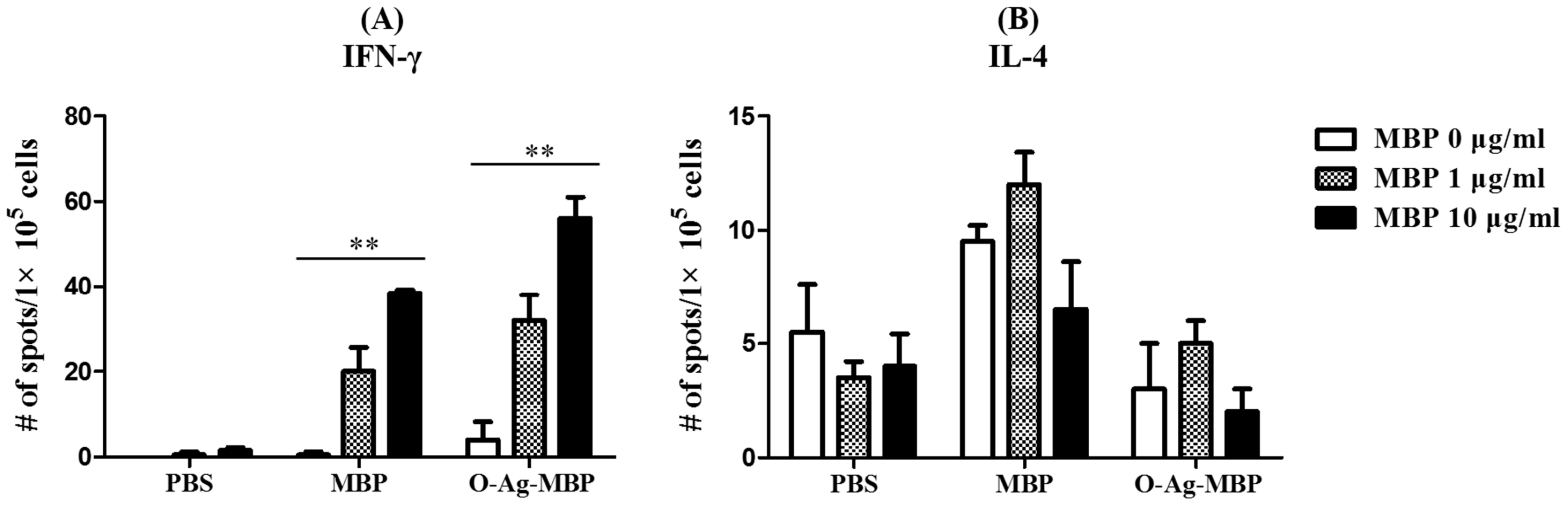
Cytokines determined by ELISPOT. (**A**), IFN-γ; (**B**), IL-4. The spots for cytokine-producing lymphocytes after stimulation with MBP or O-Ag-MBP were counted and expressed based on 1×10^5^ cells. Results are expressed as the arithmetic mean ±SD indicated by error bars. Differences of three groups stimulated at the same dose of MBP are indicated with symbols (*: P<0.05; **: P<0.001).

MBP upregulated IFN-γ and downregulated IL-4 (Figure 7A), which was consistent with previous demonstrations that MBP induced Th1-biased responses [10]. To further determine whether MBP as carrier protein of O-Ag-MBP induced Th1 activation, seven days after the third immunization, splenic mononuclear cells were collected and incubated with different doses of MBP (0 µg/ml, 1 µg/ml or 10 µg/ml) in vitro for 24 hrs, and IFN-γ and IL-4 secretions were assessed by ELISPOT. When the cells were stimulated with MBP, the number of IFN-γ producing cells increased in both MBP (P<0.01) and O-Ag-MBP (P<0.01) groups in a dose-dependent manner (Figure 8A). These results demonstrated that MBP could activate Th1 cells no matter MBP was immunized alone (MBP group) or linked to O-Ag (O-Ag-MBP group). On the contrary, when the cells were stimulated with MBP, the number of IL-4 producing cells decreased in both MBP and O-Ag-MBP groups when the doses of MBP was increased from 1 µg/ml to 10 µg/ml (Figure 8B), but the differences elicited by different doses of MBP was not significant.

## Discussion

The developments of glycoconjugate vaccines were remarkable over the last 30 years [25]. Whereas, the syntheses via traditional chemical strategies always suffer variable batch-to-batch composition, difficult quality control, inconsistent potency and high production cost [16]. An inexpensive and qualified method is desperately required. In this study, we demonstrated the production of *E. coli* O157:H7 O-Ag conjugate via a novel bacterial protein N-linked glycosylation system. Our glycoconjugate O-Ag-MBP was homogenous, and no *E. coli* O157:H7 for polysaccharide extraction and no toxic chemical reagents for chemical linkage are required during its expression and purification, which would be safe as vaccines towards mice or even humans. In addition, we showed that *E. coli* O157:H7 O-Ag could serve as substrate for *C. jejuni* OTase PglB, which provided another evidence for the relaxed substrate specificity of PglB.

MBP could serve as an ideal carrier protein of glycoconjugate vaccines for several advantages. Firstly, MBP does not have to carry an extra signal sequence, because MBP has its own signal peptide (known as MalE) to locate MBP to periplasm [26] (PglB-mediated protein glycosylation reaction takes place in periplasm of *E. coli*); Secondly, MBP augments the antigenicity of the target protein by facilitating the interactions between the antigen and antigen-presenting cells (APCs) and/or binding and transporting the antigen to secondary lymphoid organs [27]; Lastly, MBP shows potential as a TLR4 agonist to promote Th1-type responses and associated IgG2 antibody, and elicits IFN-γ from spleen mononuclear cells. [10]. In this work, we initiated the exploration of MBP as a carrier protein in glycoconjugate vaccines. Our findings demonstrated that the modified MBP carrying four consecutive N-glycosylation motifs GT at C-terminal could be successfully expressed and conjugated with *E. coli* O157:H7 O-Ag. In addition, O-Ag-MBP also promoted Th1-type responses and associated IgG2a antibody, and elicited IFN-γ from spleen mononuclear cells in response to MBP. Therefore, MBP showed TLR4 agonist-like properties to activate Th1 cells as carrier protein of O-Ag-MBP.

In previous work, *E. coli* O157:H7 O-Ag conjugated with other proteins like rEPA and Stx elicited statistically significant increases in levels of IgG and IgM towards *E. coli* O157:H7 LPS [13], [14]. Meanwhile, little work reported the secretory of IgA (sIgA) in glycoconjugate vaccine. In our work, O-Ag-MBP elicited high titers of serum bactericidal antibodies including IgG and IgM. Meanwhile, it was inspiring to find the expression of sIgA elicited by O-Ag-MBP, which was suggested to prevent effectively the colonization by enteric pathogens [28].

Several articles argued that humoral responses induced by *E. coli* O157:H7-specific O-Ag-cholera toxin and O-Ag-horse serum albumin failed to prevent following colonization of *E. coli* O157:H7 no matter parenteral or oral immunization of mice [29], [30]. The reason might be that cellular response was not elicited by these glycoconjugates, such cellular response was always considered to be more effective to clean pathogens [15]. In our work, the cellular response was elicited by O-Ag-MBP, with the simulation of CD8^+^ T cells, Th1-biased CD4^+^ T cells. CD8^+^ cytotoxic T lymphocytes (CTL) offer protection by complement-mediated cell lysis, resulting in the recruitment of macrophages, granulocytes, and eosinophils [31], [32]. Th1 CD4^+^ T cells can also stimulate macrophages by secreting IFN-γ.

In summary, *E. coli* O157:H7 O-Ag was a suitable substrate for PglB, and the glycoconjugate O-Ag-MBP elicited both humoral and Th1-biased cellular responses. Furthermore, MBP in O-Ag-MBP contributed a lot to the Th1-biased cellular responses of O-Ag-MBP as a TLR4 agonist, providing the first evidence of the potential role of MBP as a novel carrier protein in glycoconjugate vaccines against pathogens.

## Supporting Information

Figure S1 **Genesencoding enzymes responsible for the expression of** ***E. coli*** **O157:H7 O-Ag were assembled.** They comprise *rfb* gene cluster (responsible for the assembly, transport and polymerization of the O-Ag) and *wzz* gene (regulate the chain length of O-Ag).
(DOC)
