# Supplementary material for: Glycoconjugate Vaccine Containing Escherichia coli O157:H7 O-Antigen Linked with Maltose-Binding Protein Elicits Humoral and Cellular Responses
Source: PLoS One. 2014 Aug 19;9(8):e105215. doi: 10.1371/journal.pone.0105215 (PMC4138118; doi:10.1371/journal.pone.0105215)
Supplement: Figure S1 — Genesencoding enzymes responsible for the expression of E. coli O157:H7 O-Ag were assembled. They comprise rfb gene cluster (responsible for the assembly, transport and polymerization of the O-Ag) and wzz gene (regulate the chain length of O-Ag). (DOC) [file pone.0105215.s001.doc]

# Supporting Information

# for

**Glycoconjugate Vaccine Containing *Escherichia coli* O157:H7 O-antigen Linked with Maltose-binding Protein Elicits Humoral and Cellular Responses**

Zhongrui Ma^1^, Huajie Zhang^1^, Wenjing Shang^2^, Faliang Zhu^3^, Weiqing Han^4^, Xueer Zhao^1^, Donglei Han^1^, Peng George Wang^1^, Min Chen^1^*

*1 The State Key Laboratory of Microbial Technology and School of Life Sciences, National Glycoengineering Research Center, Shandong University, Jinan, Shandong, China*

*2 Department of Biochemistry and Molecular Biology, Binzhou Medical University, YanTai, Shandong, China*

*3 Department of Immunology, Shandong University School of Medicine, Jinan, Shandong, China*

*4. College of Pharmacy and the State Key Laboratory of Medicinal Chemical Biology, Nankai University, Tianjin, China*

**corresponding author*

*chenmin@sdu.edu.cn, 86 (531) 88366078*

# List of Figures

Figure S1. Genes encoding enzymes responsible for the expression of *E. coli* O157:H7 O-Ag were assembled.


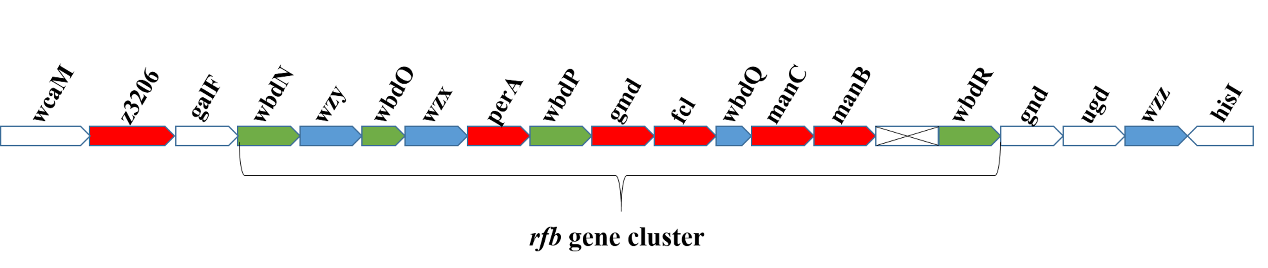


**Figure S1. Genesencoding enzymes responsible for the expression of *E. coli* O157:H7 O-Ag were assembled.** They comprise *rfb* gene cluster (responsible for the assembly, transport and polymerization of the O-Ag) and *wzz* gene (regulate the chain length of O-Ag).
